# Supplementary material for: POCKET-seq enables genome-wide profiling of on- and off-target transcriptional regulation events by dCas9-KRAB during CRISPR interference experiments
Source: bioRxiv. 2026 Jun 12:2026.06.01.729390. Originally published 2026 Jun 2. Preprint. [Version 2] doi: 10.64898/2026.06.01.729390 (PMC13252386; doi:10.64898/2026.06.01.729390)

bioRxiv preprint doi: https://doi.org/10.64898/2026.06.01.729390; this version posted June 12, 2026. The copyright holder for this preprint (which was not certified by peer review) is the author/funder, who has granted bioRxiv a license to display the preprint in perpetuity. It is made available under a CC-BY 4.0 International license.

Supplemental Figure 1:

A. Representative microscopy images showing mVenus-PTS (fluorescent reporter) and PMP70 (immunofluorescence staining) in CRISPRi non-targeting control (NTC) and *SOCS3*-depleted (SOCS3_g1 and SOCS3_g4) HCT116 cells. Data shown are generated from at least m = 24 images and n = 500 cells. Scale bar represents 10 µm.
B. Quantification of mVenus-PTS foci area from panel S1A as a percentage of cell area. The p values were derived from an independent t-test. *ns*, not significant; ****$p \leq 0.0001$.
C. Quantification of mVenus-PTS foci per cell from panel S1A. The p values were derived from an independent t-test. *ns*, not significant; ****$p \leq 0.0001$.
D. RT-qPCR for *SOCS3* mRNA levels in CRISPRi non-targeting control (NTC) and *SOCS3*-depleted (SOCS3_g1 and SOCS3_g4) HCT116 cells. Data represented as the fold change of *SOCS3* transcript levels normalized to NTC. Data shown are representative of n = 3 biological replicates.
E. Quantification of mVenus-PTS foci area as a percentage of cell area from live cell microscopy of mVenus-PTS (fluorescent reporter) in CRISPRi non-targeting control (NTC) and *SOCS3*-depleted (SOCS3_g1) HCT116 cells with either no addback or addback of *SOCS3* cDNA. Addbacks are stably expressed under hEF1a promoters. Data shown are generated from at least m = 32 images and n = 1500 cells. The p values

bioRxiv preprint doi: https://doi.org/10.64898/2026.06.01.729390; this version posted June 12, 2026. The copyright holder for this preprint (which was not certified by peer review) is the author/funder, who has granted bioRxiv a license to display the preprint in perpetuity. It is made available under a CC-BY 4.0 International license.

were derived from an independent t-test. ***$p \leq 0.001$. Representative microscopy images can be found in S2C.

Supplemental Figure 2:

A. Raw western blots of input (IN) and anti-HA tag immunoprecipitation (IP) fractions during the POCKET-seq ChIP procedure from CRISPRi non-targeting control (NTC) and *SOCS3*-depleted (SOCS3_g1 and SOCS3_g4) HCT116 cells where dCas9-KRAB is epitope tagged with HA. From left to right, blots show total protein (Stain Free), protein ladder (Colorimetric), anti-HA tag signal (Chemiluminescence), and merge (Composite).
B. RT-qPCR for *PEX19* mRNA levels in CRISPRi non-targeting control (NTC) and *SOCS3*-depleted (SOCS3_g1 and SOCS3_g4) HCT116 cells. Data represented as the fold change of *PEX19* transcript levels normalized to NTC. Data shown are representative of $n = 3$ biological replicates.
C. Representative live cell microscopy images showing mVenus-PTS (fluorescent reporter) in CRISPRi non-targeting control (NTC) and *SOCS3*-depleted (SOCS3_g1) HCT116 cells with either no addback, addback of *PEX19* cDNA, or addback of *SOCS3* cDNA. Addbacks are stably expressed under hEF1a promoters. Data shown are generated from at least $m = 32$ images and $n = 1500$ cells.
F. Quantification of total mVenus-PTS foci intensity over total cytoplasmic mVenus-PTS intensity from panel S2C. The p values were derived from an independent t-test. ***$p \leq 0.001$; ****$p \leq 0.0001$.

Supplementary Information

Additional file 1. Supplementary Figures S1-S2.

bioRxiv preprint doi: https://doi.org/10.64898/2026.06.01.729390; this version posted June 12, 2026. The copyright holder for this preprint (which was not certified by peer review) is the author/funder, who has granted bioRxiv a license to display the preprint in perpetuity. It is made available under a CC-BY 4.0 International license.

Supplemental Figure 1

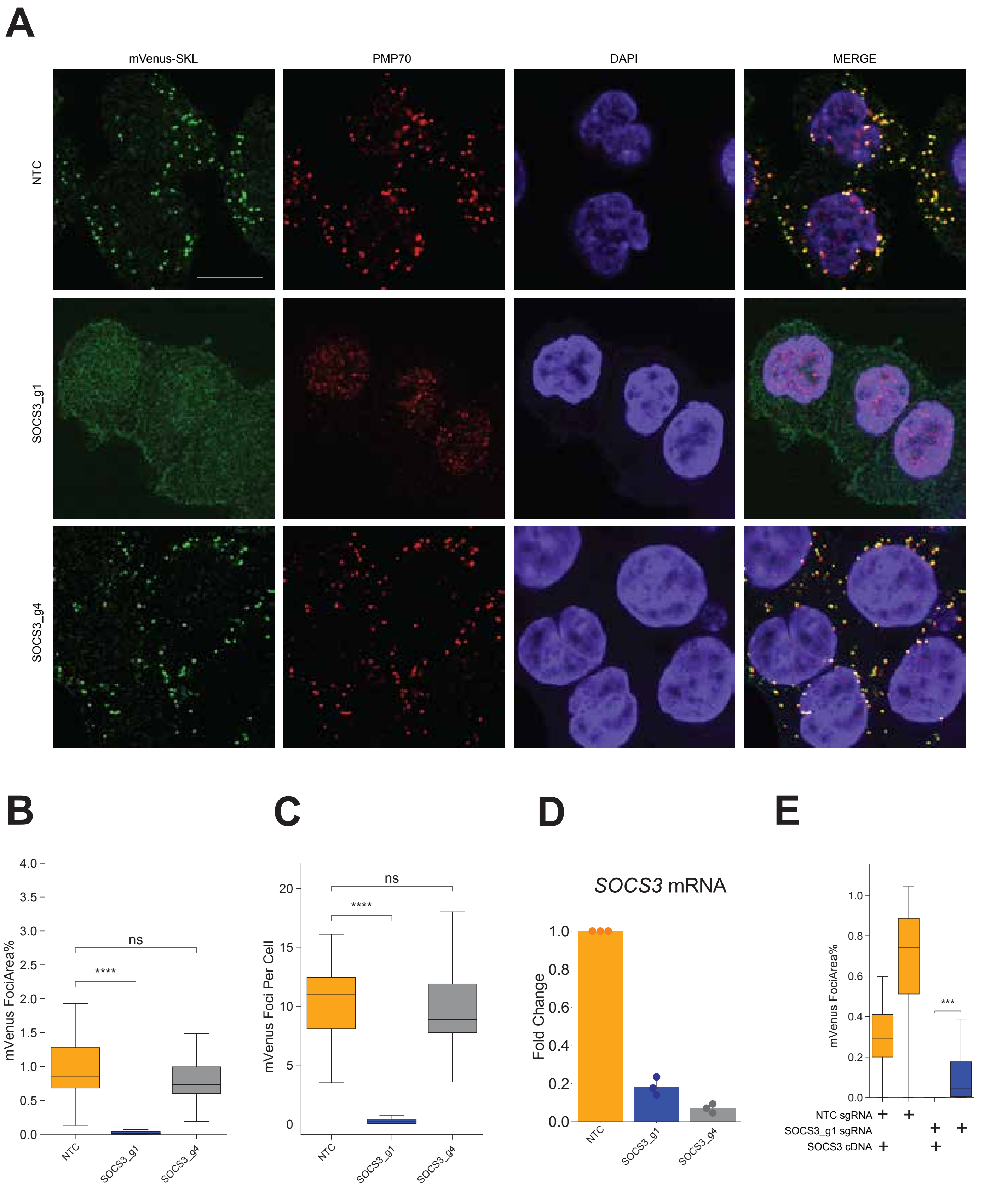

bioRxiv preprint doi: https://doi.org/10.64898/2026.06.01.729390; this version posted June 12, 2026. The copyright holder for this preprint (which was not certified by peer review) is the author/funder, who has granted bioRxiv a license to display the preprint in perpetuity. It is made available under a CC-BY 4.0 International license.

Supplemental Figure 2

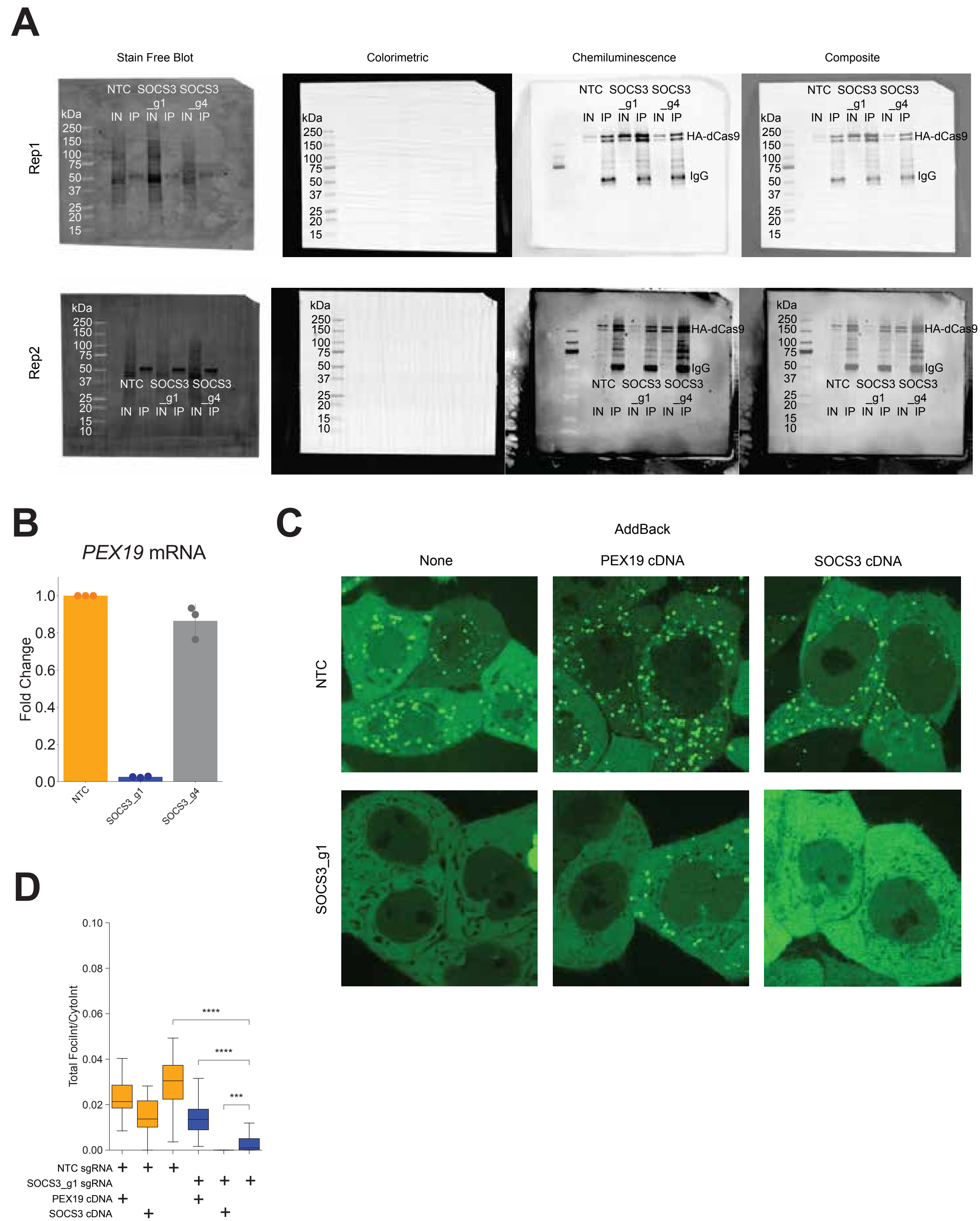

Supplement: Supplementary file 1 [file NIHPP2026.06.01.729390v2-supplement-1.pdf]
