# Supplementary material for: POCKET-seq enables genome-wide profiling of on- and off-target transcriptional regulation events by dCas9-KRAB during CRISPR interference experiments
Source: bioRxiv. 2026 Jun 2:2026.06.01.729390. Preprint. [Version 1] doi: 10.64898/2026.06.01.729390 (PMC13252386; doi:10.64898/2026.06.01.729390)

bioRxiv preprint doi: https://doi.org/10.64898/2026.06.01.729390; this version posted June 2, 2026. The copyright holder for this preprint (which was not certified by peer review) is the author/funder, who has granted bioRxiv a license to display the preprint in perpetuity. It is made available under a CC-BY 4.0 International license.

Supplemental Figure 1:

A. Representative microscopy images showing mVenus-PTS (fluorescent reporter) and PMP70 (immunofluorescence staining) in CRISPRi non-targeting control (NTC) and *SOCS3*-depleted (SOCS3_g1 and SOCS3_g4) HCT116 cells. Data shown are generated from at least *m* = 24 images and *n* = 500 cells. Scale bar represents 10 µm.
B. Quantification of mVenus-PTS foci area from panel S1A as a percentage of cell area. The *p* values were derived from an independent t-test. *ns*, not significant; *****p* ≤ 0.0001.
C. Quantification of mVenus-PTS foci per cell from panel S1A. The *p* values were derived from an independent t-test. *ns*, not significant; *****p* ≤ 0.0001.
D. RT-qPCR for *SOCS3* mRNA levels in CRISPRi non-targeting control (NTC) and *SOCS3*-depleted (SOCS3_g1 and SOCS3_g4) HCT116 cells. Data represented as the fold change of *SOCS3* transcript levels normalized to NTC. Data shown are representative of *n* = 3 biological replicates.
E. Quantification of mVenus-PTS foci area as a percentage of cell area from live cell microscopy of mVenus-PTS (fluorescent reporter) in CRISPRi non-targeting control (NTC) and *SOCS3*-depleted (SOCS3_g1) HCT116 cells with either no addback or addback of *SOCS3* cDNA. Addbacks are stably expressed under hEF1a promoters. Data shown are generated from at least *m* = 32 images and *n* = 1500 cells. The *p* values

bioRxiv preprint doi: https://doi.org/10.64898/2026.06.01.729390; this version posted June 2, 2026. The copyright holder for this preprint (which was not certified by peer review) is the author/funder, who has granted bioRxiv a license to display the preprint in perpetuity. It is made available under a CC-BY 4.0 International license.

were derived from an independent t-test. ***$p \leq 0.001$. Representative microscopy images can be found in S2C.

Supplemental Figure 2:

A. Raw western blots of input (IN) and anti-HA tag immunoprecipitation (IP) fractions during the POCKET-seq ChIP procedure from CRISPRi non-targeting control (NTC) and *SOCS3*-depleted (SOCS3_g1 and SOCS3_g4) HCT116 cells where dCas9-KRAB is epitope tagged with HA. From left to right, blots show total protein (Stain Free), protein ladder (Colorimetric), anti-HA tag signal (Chemiluminescence), and merge (Composite).
B. RT-qPCR for *PEX19* mRNA levels in CRISPRi non-targeting control (NTC) and *SOCS3*-depleted (SOCS3_g1 and SOCS3_g4) HCT116 cells. Data represented as the fold change of *PEX19* transcript levels normalized to NTC. Data shown are representative of $n = 3$ biological replicates.
C. Representative live cell microscopy images showing mVenus-PTS (fluorescent reporter) in CRISPRi non-targeting control (NTC) and *SOCS3*-depleted (SOCS3_g1) HCT116 cells with either no addback, addback of *PEX19* cDNA, or addback of *SOCS3* cDNA. Addbacks are stably expressed under hEF1a promoters. Data shown are generated from at least $m = 32$ images and $n = 1500$ cells.
F. Quantification of total mVenus-PTS foci intensity over total cytoplasmic mVenus-PTS intensity from panel S2C. The p values were derived from an independent t-test. ***$p \leq 0.001$; ****$p \leq 0.0001$.

Supplementary Information

Additional file 1. Supplementary Figures S1-S2.

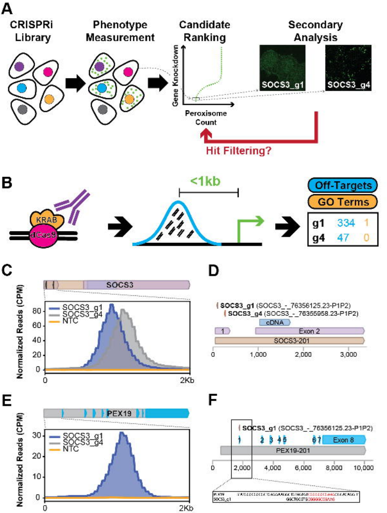
A
CRISPRi Library
Phenotype Measurement
Candidate Ranking
Secondary Analysis
Gene Knockdown
Peroxisome Count
SOCS3_g1
SOCS3_g4
Hit Filtering?
B
KRAB
dCas9
<1kb
Off-Targets
GO Terms
g1 334 1
g4 47 0
C
SOCS3
Normalized Reads (CPM)
SOCS3_g1
SOCS3_g4
NTC
80
60
40
20
0
0
2Kb
D
SOCS3_g1 (SOCS3_-_76356125.23-P1P2)
SOCS3_g4 (SOCS3_-_76355958.23-P1P2)
cDNA
1
Exon 2
SOCS3-201
0
1,000
2,000
3,000
E
PEX19
Normalized Reads (CPM)
SOCS3_g1
SOCS3_g4
NTC
30
20
10
0
0
2Kb
F
SOCS3_g1 (SOCS3_-_76356125.23-P1P2)
1
2 3 4 5
6 7
Exon 8
PEX19-201
0
2,000
4,000
6,000
8,000
10,000

A

Name	# of Off-Targets	Chr	Start	End	Sequence
SOCS3_g1	334	17	76,356,104	76,356,126	CCCTCCCTGCGGGGCCCGAAGCGG
SOCS3_g4	47	17	76,355,937	76,355,959	CCCGCCCGTGAAGTCCACAAAGG
VEGFA_2	40	6	43,730,570	43,730,556	GACCCCCTCCACCCCGCCTCCGG
HEK293_s4	3429	20	31,349,756	31,349,778	GGCACTGCGGCTGGAGGTGGTGG

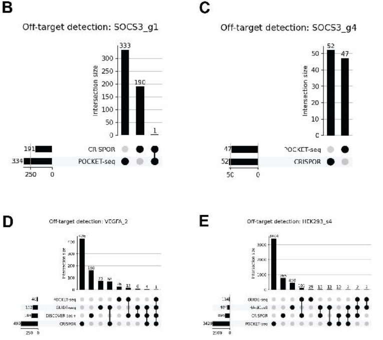

Supplement: Supplementary file 1 [file NIHPP2026.06.01.729390v1-supplement-1.pdf]
